# Clinical Remarks on Certain Diseases of the Eye, and on Miscellaneous Subjects, Medical and Surgical, Including Gout, Rheumatism, Fistula, Cancer, Hernia, Indigestion, &c. &c.

**Published:** 1843-07

**Authors:** 


Art. VI.—*Clinical Remarks on certain Diseases of the Eye, and on Miscellaneous Subjects, Medical and Surgical, including Gout, Rheumatism, Fistula, Cancer, Hernia, Indigestion, &c. &c.* By John Charles Hall, m.d. of East Retford.—*London*, 1843. 8vo, pp. 228.

Dr. Hall appears to have followed the plan of keeping a common-place-and-case-book, in which he has entered, from time to time, such extracts from the authors he has perused, on a variety of professional subjects, as struck him to be interesting, along with such cases, occurring in his practice, as seemed to be remarkable. Nothing can be more laudable than thus recording the results of his reading and experience, on diseases of the eye, gout, rheumatism, fistulæ, cancer, hernia, indigestion, &c., but the propriety of publishing such a collection of extracts and notes of cases, may fairly be doubted.

As a sample of the work, let us take chapter viii. It consists of two cases of uterine hemorrhage, in which the placenta was situated over the os uteri; the one treated by the author, by introducing the hand, and turning, and the other by some other practitioner not named, managed in a similar way, except that turning was not required as the breech presented; to which two cases are added a third, in which the result was fatal, copied from Dr. Merriman's works, along with scraps from Burns, Lee, and other well-known authors. Now, this is very good as a specimen of Dr. Hall's common-place-book, very useful for him to refer to, when similar cases occur in his practice, but possessing neither interest nor novelty sufficient to be put into print.

The coming before the public as an author is a very serious step; for if the experiment is premature, unnecessary, or without value, a published work can only be referred to as a proof of ill-judged forwardness on the part of the author. Dr. Hall, we doubt not, is deficient neither in powers of observation nor in those of reflection. Far better then, to wait a few years, exercise those powers in the various interesting professional subjects which might come before him, and then give to the public the well-concocted result, than to offer them such a collection of shreds as the present.

Rather more than the first half of the volume is devoted to diseases of the eye, the treatment recommended being on the whole judicious. Dr. Hall entertains a high opinion of mercury, and especially of the bi-chloride, an alterative, which, he says, "is not, even yet, used so extensively as it ought."

> "It can be given in solution, which is a considerable advantage, rendering its action, much more certain, more equal, and by readier absorption, probably more effectual in producing an alterative influence upon the whole system." (p. 14.)
>
> "It is also worthy of note that this medicine may be continued, in uninterrupted use, for a very considerable period, without obvious injury or inconvenience, and in certain cerebral or spinal disorders, a long unbroken course of this preparation is of singular avail." (p. 15.)

The following remarks on opacities of the cornea are judicious:

> "It is very important to distinguish, as nearly as possible, those opacities of the cornea which are likely to be removed by the unaided efforts of nature, from those which cannot be dispersed without the assistance of art, and also to ascertain the proper season for commencing the use of remedies, for, if there be any external inflammation, an irritable state of the eye or of the general health, or if the opacity be the result of chronic corneitis, it would be unadvisable to apply local stimulants until these affections are removed." (p. 59.)

We quite agree with Dr. Hall, in the ridicule and contempt he throws on the squint-clippers, who, "without experience, without reflection, without a day's examination of the merits or results of the operation, positively employed their pupils and others to kidnap patients into their operating room." (p. 97.) The ignorance of some of those persons was equalled only by their impudence and brutality. Dr. Hall does not appear to have been very successful in cutting for strabismus. The cause of this appears to be his confining the operation generally to the worst eye only, and leaving the less affected eye untouched.

Dr. Hall is of opinion that the operations for cataract are more hazardous during cold and damp weather. He supports this notion by the authority of Mr. Tyrrell, who, he says, operated for extraction only from October to March. Surely, this is a typographical mistake.

On points of history, Mr. Hall is generally wrong; as, for instance, where he ascribes the notion, that in purulent ophthalmia the cornea dies from the pressure caused by the chemosed conjunctiva, to Mr. Middlemore, (p. 56;) the first demonstration of the advantage of mercury in iritis, to Dr. Farre, (p. 101;) &c. &c.

Dr. Hall tells us, that he has no intention to lay claim to anything that is new in the treatment of fistula. Then, why trouble us with a school-boy treatise, made up of the opinions of Pott, Samuel Cooper, Brodie, Liston, and Roux?

No sound reason is offered for the following extraordinary dictum, regarding the operation for cancer of the breast. Dr. Hall speaks unfavorably of the success of the operation, being of opinion that in the great majority of cases the disease reappears. "Yet, it is our duty to operate," says he, "even under unfavorable circumstances." (p. 181.)

